# Tart Cherry Extract Containing Chlorogenic Acid, Quercetin, and Kaempferol Inhibits the Mitochondrial Apoptotic Cell Death Elicited by Airborne PM_10_ in Human Epidermal Keratinocytes

**DOI:** 10.3390/antiox10030443

**Published:** 2021-03-13

**Authors:** Do-Wan Kim, Dae-Hwa Jung, Junghee Sung, In Sun Min, Sei-Jung Lee

**Affiliations:** 1 Department of Pharmaceutical Engineering, Daegu Haany University, Gyeongsan 38610, Korea; sosr200211@gmail.com (D.-W.K.); jdh8024@hanmail.net (D.-H.J.); 2 R&D Center, Reanzen Co. Ltd., Anyang 14056, Korea; sky1231002@naver.com; 3 Fragrance of the Moon, 23 Taepyeong-ro, Jung-gu, Daegu 41900, Korea; etuire0209@gmail.com

**Keywords:** apoptosis, human epidermal keratinocytes, particulate matter, tart cherry

## Abstract

Tart cherry (*Prunus cerasus* L.), a medicinal food containing high concentrations of phytochemicals, has a variety of antioxidant activities and health benefits. Here, we investigate the functional effect of tart cherry during apoptotic cell death elicited by airborne particulate matter with a diameter of <10 μm (PM_10_) in human epidermal keratinocyte HaCaT cells. The PM_10_ particles significantly induced cytotoxicity in the HaCaT cells. The decrease in cell viability was restored upon treatment with tart cherry extract (200 μg/mL) containing chlorogenic acid, quercetin, and kaempferol. Tart cherry inhibited the intracellular reactive oxygen species (ROS) responsible for the distinctive activations of the extracellular signal-regulated kinase (ERK) and p38 mitogen-activated protein kinase (MAPK) in PM_10_-treated HaCaT cells. Interestingly, tart cherry significantly inhibited the expression of apoptosis-related genes (B-Cell Lymphoma 2 (Bcl-2), Bcl-2 associated X protein (Bax), and caspase-3) as regulated by the activation of transcription factor nuclear factor-kappa B (NF-κB). These results demonstrate that tart cherry is a medicinal food that blocks the mitochondrial pathway of apoptosis induced by PM_10_ in human epidermal keratinocytes.

## 1. Introduction

Fine particulate matter (PM_10_), referring to particles with an aerodynamic diameter of less than 10 μm, mainly consists of air pollutants in the form of a fine dust (FD) that can contain metals, organic toxic compounds, and biological materials depending on the natural and the anthropogenic dust source [1]. PM is primarily formed from the chemical reactions triggered by exhaust gases from internal combustion engines, smoke from factory chimneys, and fuel combustion in the atmosphere [2]. This fine dust can have various adverse effects on humans’ health and on the ecosystem [1]. For instance, PMs can cause numerous health problems that are associated with respiratory, cardiovascular, and skin diseases [3].

Skin, the primary tissue exposed to FD, plays an important role as an interface between air pollutants and the body. However, it was proven that PM is able to be absorbed by the skin barrier and causes many inflammatory and allergic reactions and delays the skin wound healing process induced by topical exposure [4]. Keratinocytes are a major cell type in the epidermis, which is the outermost layer of the skin and which serves mainly to protect against microbial, viral, fungal, and ambient forms of air pollution as a major skin barrier [5]. Recent studies suggest that keratinocytes, when exposed to PM, induce the mitochondrial apoptosis pathway by producing reactive oxygen species (ROS) to interact with intracellular lipids, proteins, and nucleic acids, leading to chromatin condensation, membrane blebbing, cell shrinkage, and the formation of apoptotic bodies.

Indeed, keratinocyte apoptosis plays a critical role in a number of phenomena of the skin, including premature aging of the skin [6] and inflammatory diseases such as atopic dermatitis, acne, and psoriasis [7]. Given that patients with compromised epidermal barriers are more susceptible to PM through its increased absorption [4], it is crucial to investigate pharmacological substances that regulate the oxidative stress pathway of PM in keratinocytes to maintain the integrity of the skin barrier. Thus far, investigations pertaining to the impact on the skin by PM are far less abundant as compared to those pertaining to the respiratory and cardiovascular systems.

Tart cherry, a species of *Prunus* in the subgenus Cerasus (*Prunus cerasus* L.), is widely distributed in Europe and southwest Asia. Tart cherry has received attention due to its high content of anthocyanin-related polyphenolics compared to other varieties of cherries and fruits, and for its possible human health benefits [8]. In addition, recent studies have shown that tart cherry has various therapeutic effects such as antioxidant, anti-inflammatory [9], and muscle recovery effects [10] and that it is useful as a treatment for insomnia [11]. While tart cherry has been shown to have attractive therapeutic efficacy as a treatment for many diseases, its role in skin pathogenesis as induced by FD remains unclear. In this study, therefore, we investigate the functional role of tart cherry during apoptotic cell death elicited by fine particulate matter in human epidermal keratinocytes and the mechanism underlying the beneficial effects of tart cherry with regard to the skin barrier function.

## 2. Materials and Methods

### 2.1. Chemicals

Fetal bovine serum (FBS) was purchased from GE Healthcare (Logan, UT, USA). Fine dust (PM_10_-like, European reference material ERM-CZ120) was obtained from Sigma-Aldrich (St. Louis, MO, USA). Turkish 100% not from concentrate (NFC) Montmorency tart cherry (*Prunus cerasus* L.) juice was purchased from KNF KOREA INC (Gangseo-gu, Busan, Korea). Extracellular signal-regulated kinase (ERK), p-ERK, p38, p-p38, nuclear factor-kappa B (NF-κB)p65, p-NF-κBp65, c-Jun *N*-terminal kinases (JNK), p-JNK, B-Cell Lymphoma 2 (Bcl-2), Bcl-2 associated X protein (Bax), cleaved caspase-3, and β-actin antibodies were purchased from Santa Cruz Biotechnology (Paso Robles, CA, USA). Goat anti-rabbit and -mouse IgG (HRP) antibodies were purchased from Abcam (Cambridge, MA, USA). *N*-acetylcysteine (NAC) was obtained from Tocris (Minneapolis, MN, USA). PD98059, SB203580, and Bay 11-7082 were purchased from Med Chem Express (Monmouth Junction, NJ, USA), and 5-(and-6)-chloromethyl-2′,7′-dichlorodihydrofluorescein diacetate, acetyl ester (CM-H_2_DCFDA) was obtained from Invitrogen (Carlsbad, CA, USA). All chemicals used were of analytical grade and were used as received without any further purification and were obtained from Sigma-Aldrich (St. Louis, MO, USA).

### 2.2. Preparation of the Tart Cherry Extraction

Turkish 100% NFC (not from concentrate) Montmorency tart cherry (*Prunus cerasus* L.) juice was purchased in August 2020 from KNF KOREA INC (Gangseo-gu, Busan, Korea). The NFC tart cherry juice (5 L) was filtered using Whatman filter paper (No. 2) to remove debris and then concentrated with an evaporator (N1000; EYELA, Tokyo, Japan). Next, the enriched solution of tart cherry was dried with a freeze-dryer (FDB-5503; Operon, Gyeonggi-do, Korea). The resulting crude extracts were stored at −20 °C until further use.

### 2.3. Ultra Performance Liquid Chromatography (UPLC)

Ultra performance liquid chromatography (UPLC) analyses were carried out in a Waters^®^ Acquity UPLC system equipped with a photodiode array (PDA) detector, an auto sampler, and a column oven (Waters, Prague, Czech Republic). Chromatographic separation was achieved using a Waters^®^ Acquity UPLC BEH C_18_ column (2.1 × 100 mm i.d., 1.7 μm) at 37 °C. To prepare the test liquid for a quantitative analysis, tart cherry extract (1 mg) was mixed with 10 mL of 30% methanol and extracted using an ultrasonic microwave extractor (Powersonic 505; Hwashin Tech, Daegu, Korea) for 1 h. The test liquid was filtered using a membrane filter with a diameter of <0.2 μm. On the other hand, proper amounts of internal standards (chlorogenic acid, quercetin, and kaempferol) were measured accurately and dissolved in methanol to obtain stock solutions at a concentration of 1 mg/mL. Each solution was diluted in methanol to obtain working solutions with standard at concentrations of 12.5, 25, 50, and 100 μg/mL. The analysis conditions are presented in Table 1. The standard curve determination coefficient (R^2^) value of all standard materials exceeded 0.999. The injection volume was 2 μL and the flow rate was kept constant at 0.4 mL/min. The mobile phase consisted of a 13-min gradient system combining water and acetonitrile containing 0.1% aqueous formic acid (FA). Data were processed using the Empower 3 (Waters^®^) software (Waters, Prague, Czech Republic). The chromatograms detected by UPLC-PDA (Waters, Prague, Czech Republic) were recorded at a wavelength of 330 nm for chlorogenic acid and quercetin and at 380 nm for the kaempferol. Peaks were identified by comparing retention times and were quantitated by reference standards.

### 2.4. Culture of Human Skin Keratinocyte and Inhibitor Treatments

Human epidermal keratinocyte HaCaT cells were obtained from the American Type Culture Collection (ATCC^®^, Manassas, VA, USA) and cultured at 37 °C in an incubator supplied with 5% CO_2_ in Dulbecco’s Modified Eagle’s medium (DMEM) cell culture medium (Invitrogen Co., Carlsbad, CA, USA) treated with FBS (10%), streptomycin (100 mg/mL), and penicillin (100 U/mL). The medium was replaced twice a week. All the cells were serum-starved for 24 h before treatment with tart cherry and PM_10_ in DMEM without FBS. To determine the relevance of signaling molecules in the apoptotic cell death pathway induced by PM_10_, HaCaT cells were pretreated with *N*-acetylcysteine (NAC, 10 μM), SB203580 (10 μM), PD98059 (10 μM), and Bay 11-7082 (10 μM) for the inhibition of ROS, p38 mitogen-activated protein kinase (MAPK), ERK, and NF-κB for 30 min prior exposure to PM_10_, respectively.

### 2.5. Detection of Intracellular Reactive Oxygen Species (ROS)

Cells were treated with 10 mM of CM-H_2_DCFDA for 30 min to quantify the level of intracellular ROS. After two washes with PBS, cells were scraped and loaded into a black 96-well plate. The fluorescence, which corresponds to the amount of intracellular ROS, was determined using a microplate reader designed for the detection of fluorescent and luminescent signals (SPARK, Seestrasse, Männedorf, Switzerland) at excitation and emission wavelengths of 485 and 535 nm.

### 2.6. Western Blot Analysis

The expression and phosphorylation of proteins related to the intracellular signaling pathway were determined by Western blot analysis and performed as previously described [12]. The protein bands transferred to a polyvinylidene fluoride membrane were detected and quantified using the Chemi Doc™ XRS + System (Bio-Rad, Hercules, CA, USA).

### 2.7. Live/Dead Assay and Immunofluorescence Analysis

HaCaT cells treated with PM_10_ and tart cherry were labeled with a live/dead cell assay kit (Abcam, Cambridge, MA, USA) according to the manufacturer’s protocol. Live cells with esterase activity (green) and dead cells compromising plasma membranes (red) were directly counted per random microscopic field and the numbers were converted to a percentage by multiplying by 100. Ten random fields per coverslip were counted. Nuclear translocalization of NF-κB was determined by immunofluorescence staining as previously described [12]. Cells incubated with phospho-NF-κB antibody were labeled with goat anti-rabbit IgG/IgM (H + L) (Invitrogen Co., Carlsbad, CA, USA) and counterstained with 4′,6-diamidino-2-phenylindole (DAPI) in 5% normal goat serum. The immunofluorescence signals were detected using an Olympus FluoView™ 300 confocal microscope (Center Valley, PA, USA) with 400× objective.

### 2.8. Cell Viability Assay

The viability of HaCaT cells treated with PM_10_ and tart cherry was determined by using the EZ-CYTOX kit (Dail-Lab Service, Seoul, Korea) as previously described [12]. Cells were treated with EZ-CYTOX master mix for 30 min. Cell viability was directly analyzed using a microplate reader (SPARK, Seestrasse, Männedorf, Switzerland) at 450 nm.

### 2.9. Annexin V Apoptosis Assay

The apoptosis of HaCaT cells was determined by using an Annexin V-fluorescein isothiocyanate (FITC) kit (Invitrogen, Carlsbad, CA, USA) to detect phosphatidylserine on the exterior surface of the cellular membrane as previously described [13]. The proportion of healthy, early apoptotic, late apoptotic, and necrotic cells was analyzed using the NucleoCounter^®^ advanced image cytometer (ChemoMetec, Gydevang, Allerod, Denmark) and NucleoView NC-3000 software (ChemoMetec, Gydevang, Allerod, Denmark, version 1.4.).

### 2.10. Statistical Analysis

Data are expressed as mean value± standard errors (S.E.). Statistical significance was determined by ANOVA, followed, in some cases, by a comparison of treatment means with a control using the Bonferroni–Dunn test in SPSS 16 software (IBM Corp, Armonk, NY, USA). *p* < 0.05 is considered significant.

## 3. Results

### 3.1. Tart Cherry Inhibits Keratinocyte Apoptosis Induced by PM_10_

**Figure 1:**
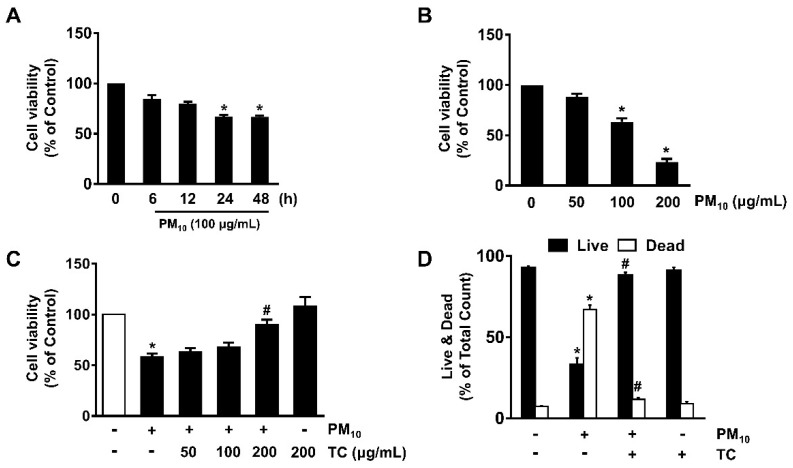
Tart cherry prevents the keratinocyte apoptosis induced by fine particulate matter (PM_10_). (**A**) Time-dependent response of PM_10_ (100 μg/mL) on cytotoxicity was determined by EZ-CYTOX assay. Data are represented as the means ± S.E. * *p* ≤ 0.05 vs. 0 h. (**B**) Cells treated with PM_10_ (0–200 μg/mL) for 24 h. Data are represented as the means ± S.E. *n* = 3. * *p* ≤ 0.05 vs. control. (**C**) Cells co-treated with tart cherry and PM_10_ for 24 h. Data are represented as the means ± S.E. *n* = 3. * *p* ≤ 0.01 vs. control. # *p* ≤ 0.01 vs. PM_10_ alone. (**D**) Cells incubated with tart cherry and PM_10_ for 24 h. Live and dead cells were determined by the live/dead assay kit. Data are represented as the means ± S.E. *n* = 3. * *p* ≤ 0.001 vs. control. # *p* ≤ 0.05 vs. PM_10_ alone.

To confirm that fine particulate matter 10 (PM_10_), which has an aerodynamic diameter of less than 10 μm, induces cytotoxicity in human epidermal keratinocytes, HaCaT cells were treated with PM_10_ at various concentrations (0–200 μg/mL) for 24 h. PM_10_ significantly induced cytotoxicity in HaCaT cells at concentrations ranging from 100 to 200 μg/mL compared to untreated cells (Figure 1A). A decrease in cell viability was observed after incubation with 100 μg/mL of PM_10_ for 24 h (Figure 1B). To investigate the protective effect of tart cherry on the cytotoxicity of fine dust, cells were treated with 100 μg/mL of PM_10_ for 24 h prior to exposure to tart cherry at various concentrations (50–200 μg/mL) for 30 min. A pretreatment of 200 μg/mL of tart cherry significantly reversed the reduced cell viability caused by PM_10_ (Figure 1C). We also determined the functional role of tart cherry to block cell death induced by PM_10_ by means of a LIVE/DEAD viability/cytotoxicity assay for simultaneous determination of live and dead cells over a period of 24 h (Figure 1D). PM_10_ significantly induced a number of dead cells, whereas for the live cells, a significant cytotoxic effect was noted. However, tart cherry significantly prevents cytotoxicity induced by PM_10_. These results indicate that the protective effect of tart cherry against fine dust exposure is related to the blocking of the death of keratinocytes caused by PM_10_.

### 3.2. Antioxidative Effect of Tart Cherry Containing Chlorogenic Acid, Quercetin, and Kaempferol in HaCaT Cells Treated with PM_10_

**Figure 2:**
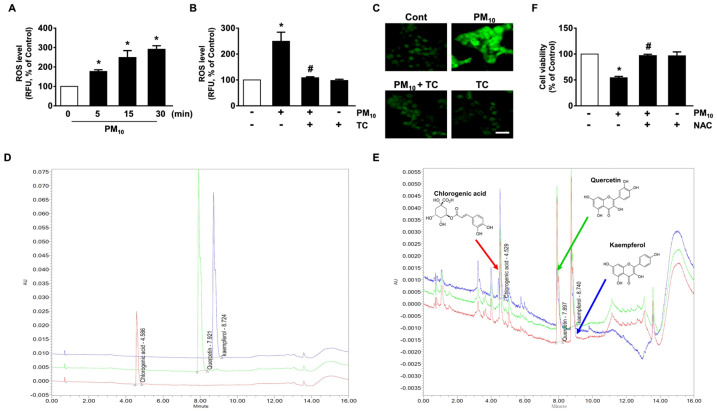
Antioxidative effect of tart cherry containing chlorogenic acid, quercetin, and kaempferol in HaCaT cells treated with PM_10_. (**A**) Time-dependent response of PM_10_ (100 μg/mL) on reactive oxygen species (ROS) production determined by staining HaCaT cells with 5-(and-6)-chloromethyl-2′,7′-dichlorodihydrofluorescein diacetate, acetyl ester (CM-H_2_DCFDA). Data are represented as the means ± S.E. *n* = 3. * *p* ≤ 0.01 vs. 0 min. (**B**) Cells treated with PM_10_ and tart cherry (TC, 200 μg/mL) for 15 min. Data are represented as the means ± S.E. *n* = 3. * *p* ≤ 0.01 vs. control. # *p* ≤ 0.001 vs. PM_10_ alone. RFU, Relative fluorescence units. (**C**) ROS production (green) visualized by confocal microscopy. Scale bars, 100 μm (original magnification × 100). *n* = 3. (**D**) Ultra performance liquid chromatography (UPLC) profile of the commercial standard compounds. (**E**) UPLC profile of three major compounds in tart cherry. The insets indicate the chemical structure of chlorogenic acid, quercetin, and kaempferol, respectively. (**F**) Cells were pretreated with the antioxidant *N*-acetylcysteine (NAC, 10 μM) for 30 min prior exposure to PM_10_ for 24 h. Cell viability was determined. Data are represented as the means ± S.E. *n* = 3. * *p* ≤ 0.001 vs. control. # *p* ≤ 0.01 vs. PM_10_ alone.

Fine dust has been shown to facilitate the production of reactive oxygen species (ROS), which results in the prominent amplification of apoptotic signals. A significant increase in the ROS level appeared between 5 and 30 min after incubation with 100 μg/mL of PM_10_ (Figure 2A), which was blocked by tart cherry treatment (Figure 2B). The inhibitory effects of tart cherry on ROS production were further visualized by staining HaCaT cells with a fluorescent dye, 2′,7′-dichlorofluorescein diacetate (CM-H_2_DCFDA) (Figure 2C). Tart cherry extracts have substantial amounts of flavonols and non-flavonoid polyphenolic compounds responsible for their antioxidant properties. To know the underlying cause of the antioxidant effects, we further investigated the contents of chlorogenic acid as a non-flavonoid polyphenol and quercetin and kaempferol as flavonols in tart cherry by mean of a UPLC analysis. The resulting chromatograms are shown in Figure 2. The concentrations of chlorogenic acid, quercetin, and kaempferol in tart cherry as determined by the calibration curves of standard compounds were 20.90 ± 0.18 mg/L at area 27,304 ± 117.5 mV × sec and height 4446 ± 19.1 mm, 8.20 ± 0.74 mg/L at area 37,875 ± 1708.5 mV × sec and height 5906 ± 266.4 mm, and 4.57.3 ± 0.12 mg/L at area 21,373 ± 280 mV × sec and height 3406 ± 44.7 mm, respectively (Table 2 and Figure 2D,E). The validation method confirmed the stability and reliability of the results, showing the consecutive separation of the three major compounds in tart cherry. On the other hand, the decrease in cell viability that occurred due to PM_10_ was significantly restored by treatment with *N*-acetylcysteine (NAC) as an antioxidant (Figure 2F). These results demonstrate that the pharmacological effect of tart cherry containing chlorogenic acid, quercetin, and kaempferol on cytotoxicity in human epidermal keratinocytes is mediated by its antioxidative potential against PM_10_.

### 3.3. Tart Cherry Inhibits the Activation of the ERK and p38 MAPK Pathways Triggered by PM_10_

**Figure 3:**
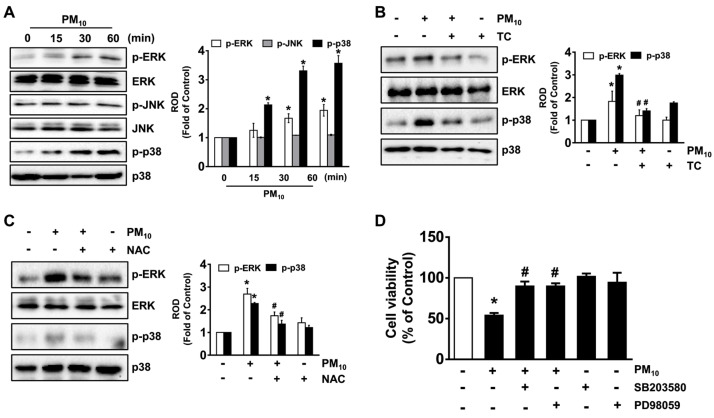
Tart cherry inhibits the activation of the extracellular signal-regulated kinase (ERK) and p38 mitogen-activated protein kinase (MAPK) pathways triggered by PM_10_. (**A**) Time-dependent response of PM_10_ (100 μg/mL) on the activation of mitogen-activated protein kinase (MAPK) in HaCaT cells determined by Western blot. (**B**) The effect of tart cherry (TC, 200 μg/mL) on the phosphorylation of ERK and p38 MAPK is shown. (**C**) Cells pre-treated with NAC prior to exposure to PM_10_ for 30 min. (**D**) Cells pretreated with SB203580 (10 μM) and PD98059 (10 μM) as inhibitors for p38 MAPK and ERK were incubated with PM_10_ for 24 h. Cell viability was determined. (**A**–**D**) Data are represented as the means ± S.E. *n* = 3. * *p* ≤ 0.01 vs. control. # *p* ≤ 0.05 vs. PM_10_ alone. ROD, relative optical density.

PM_10_ induced the phosphorylation of p38 MAPK and ERK as interesting downstream candidates for ROS production but did not affect the activation of JNK (Figure 3A), and its effect at 30 min was inhibited by treatment with tart cherry (Figure 3B), as was ROS scavenging after treatment with an antioxidant, *N*-acetylcysteine (NAC) (Figure 3C). The blockage of ERK and p38 MAPK upon treatment with PD98059 and SB203580 also significantly restored cell viability affected by PM_10_ (Figure 3D). These data indicate that the phosphorylation of ERK and p38 MAPK triggered by ROS is the critical step in the cell death signaling pathway initiated by PM_10_ and that the fine dust signaling pathway can be significantly blocked by treatment with tart cherry.

### 3.4. Tart Cherry Regulates the Activation of NF-κB Responsible for the Keratinocyte Apoptosis Caused by PM_10_

**Figure 4:**
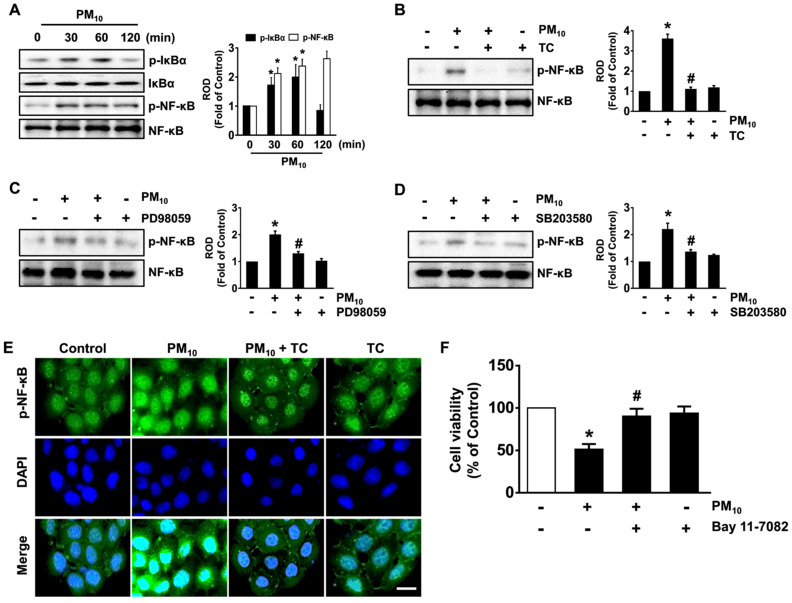
Tart cherry regulates the activation of nuclear factor-kappa B (NF-κB) responsible for the keratinocyte apoptosis caused by PM_10_. (**A**) Time-dependent response of PM_10_ (100 μg/mL) on the activation of nuclear factor of kappa light polypeptide gene enhancer in B-cells inhibitor alpha (IκBα) and NF-κB in HaCaT cells as determined by Western blot. (**B**) The effect of tart cherry (TC, 200 μg/mL) on the phosphorylation of NF-κB is shown. (**C**,**D**) Cells pretreated with PD980529 (10 μM) and SB203580 (10 μM) as inhibitors for ERK and p38 MAPK were incubated with PM_10_ for 30 min. (**E**) Representative immunofluorescence staining of NF-κB in cells co-treated with tart cherry and PM10 is shown. Scale bars, 100 μm (original magnification × 100). (**F**) Cells pretreated with Bay 11-7082 (10 μM) as an inhibitor for NF-κB were incubated with PM_10_ for 24 h. Cell viability was determined. (**A**–**F**) Data are represented as the means ± S.E. *n* = 3. * *p* ≤ 0.01 vs. control. # *p* ≤ 0.05 vs. PM_10_ alone. ROD, relative optical density.

We subsequently examined the effect of tart cherry on the activation of NF-κB mediated by the phosphorylation/degradation of nuclear factor of kappa light polypeptide gene enhancer in B-cells inhibitor alpha (IκBα) responsible for the transcriptional activation of apoptosis-related genes in keratinocytes treated with fine dust. Significant increases in the phosphorylation outcomes of IκBα and NF-κB were revealed from 30 min after treatment with 100 μg/mL of PM_10_ (Figure 4A), though the increase could be suppressed by 200 μg/mL of tart cherry (Figure 4B), the ERK inhibitor PD98059 (Figure 4C), and by the p38 MAPK inhibitor SB203580 (Figure 4D). The regulatory effect of tart cherry on the nucleic translocation of p-NF-κB induced by PM_10_ was also assessed by immunofluorescence staining (Figure 4E). The decrease in cell viability that occurred due to PM_10_ was significantly restored after incubation with the NF-κB inhibitor Bay 11-7082 (Figure 4F). Thus, these data demonstrate that tart cherry negatively regulates the activation of NF-κB potentiated by ERK and p38 MAPK, which is necessary for the apoptotic pathway triggered by PM_10_ during the promotion of keratinocytes’ cell death.

### 3.5. The Pharmacological Effect of Tart Cherry on Keratinocytes Apoptosis Induced by PM_10_

**Figure 5:**
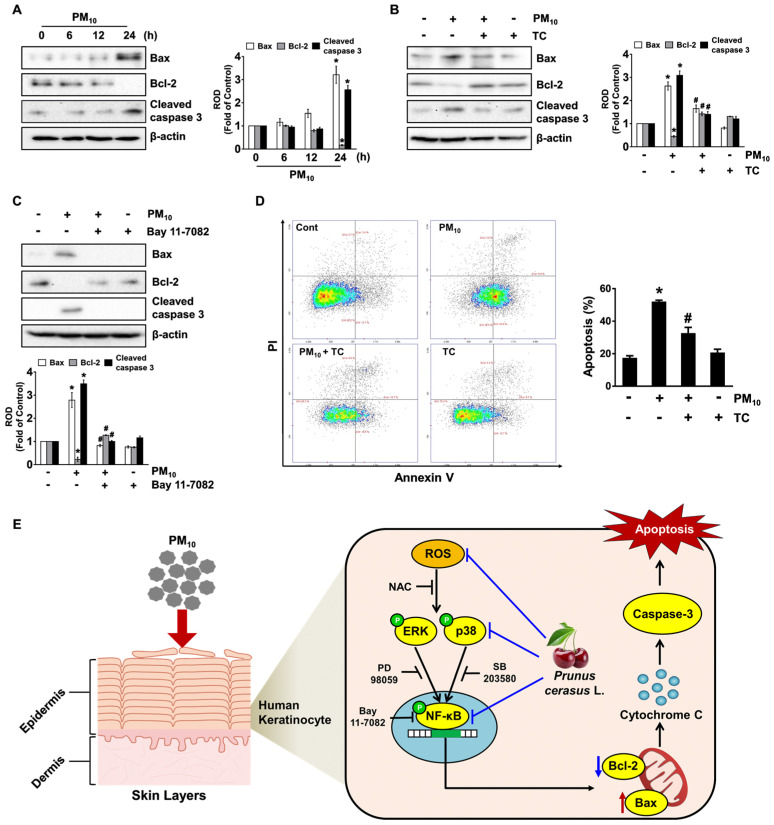
The pharmacological effect of tart cherry on keratinocyte apoptosis induced by PM_10_. (**A**) Time-dependent response of PM_10_ (100 μg/mL) on the expression of Bax, Bcl-2, and cleaved caspase-3 in HaCaT cells as determined by Western blot. (**B**) The effect of tart cherry (TC, 200 μg/mL) on the expression of apoptosis-related proteins is shown. (**C**) Cells pretreated with Bay 11-7082 (10 μM) as an inhibitor for NF-κB were incubated with PM_10_ for 24 h. (**D**) Cells were incubated with tart cherry and PM_10_ for 24 h. The proportion of apoptotic cells stained by Annexin V was analyzed using the NucleoCounter^®^ advanced image cytometer. (**E**) The sequences of putative signaling pathways are summarized. (**A**–**D**) Data are represented as the means ± S.E. *n* = 3. * *p* ≤ 0.01 vs. control. # *p* ≤ 0.01 vs. PM_10_ alone. ROD, relative optical density.

Having demonstrated the necessity of NF-κB in the regulation of keratinocyte apoptosis by PM_10_, we questioned how activated NF-κB actually coordinates with apoptosis-related proteins. PM_10_ augmented the expression of the mitochondrial pro-apoptotic regulator Bax but decreased the expression of Bcl-2 which has the anti-apoptotic function (Figure 5A), indicating that PM_10_ evokes apoptotic cell death mediated by the mitochondrial process. Moreover, PM_10_ also augmented the cleavage of caspase-3, known as an executioner caspase in apoptosis (Figure 5A). However, the increased level of the apoptosis-related proteins elicited by PM_10_ was significantly abrogated by treatment with tart cherry (Figure 5B) and NF-κB inhibitor (Figure 5C). To confirm the pharmacological effect of tart cherry in preventing the activation of the keratinocyte apoptotic signaling pathway, we subsequently undertook flow cytometric analyses over a period of 24 h by means of an Annexin V/Propidium Iodide (PI) assay for an accurate assessment of cell death outcomes (Figure 5D). PM_10_ significantly induced keratinocyte apoptosis, whereas for necrotic cell death, a marginal effect was noted. However, tart cherry significantly prevented apoptotic cell death induced by PM_10_. These results indicate that the protective effect of tart cherry on cytotoxicity in human epidermal keratinocytes is related to the blocking of apoptosis caused by PM_10_.

## 4. Discussion

In this study, we demonstrated that tart cherry can block the apoptotic signaling pathways triggered by fine particulate matter (PM) through the inhibition of MAPKs/NF-κB, a process that occurs due to ROS production in human epidermal keratinocytes (Figure 5E). Despite the wealth of evidence indicating that tart cherry has diverse biological/pharmacological effects, the anti-apoptotic mechanism of tart cherry against stressful stimuli induced by PM_10_ remained less well understood. PM_10_ is a critical air pollutant that may promote apoptotic cell death through the destruction of cellular organelles such as endoplasmic reticulum (ER), mitochondria, and lysosomes [1]. The destruction of mitochondria in skin cells causes significant pathogenesis or the progression of various diseases via ROS production, resulting in mitochondrial dysfunction and, often, in cell death [1]. In the present study, we found that PM_10_ amplifies keratinocyte apoptotic signals by producing ROS. These results are also supported by our previous findings showing that fine dust together with phthalates, a ubiquitous environmental contaminant, significantly induces programmed cell death and inflammation of epidermal keratinocytes through ROS production [14,15]. Given that keratinocytes in the skin basal layer are in close contact with extracellular matrix (ECM) components and considering the associated growth factors through integrins and receptors [16], it is important to find pharmacological substances that regulate keratinocyte apoptosis as induced by PM_10_, leading to the degradation of macromolecules in the ECM and, thus, premature skin aging [6] and inflammatory diseases [7]. Although the efficacy of tart cherry or its constituents against PM_10_ in epidermal keratinocyte cells has not been reported, many scientists insist that tart cherry contains various pharmacological substances, including polyphenol-rich contents, with numerous beneficial biological activities in the skin. In the present study, we have proven that tart cherry, which contains chlorogenic acid, quercetin, and kaempferol, has the ability to inhibit the oxidative keratinocyte apoptosis induced by PM_10_. Although identification of all of the chromatograms detected by UPLC-PDA remains a major challenge to us in our further research, tart cherry extract might be included in many functional phytochemicals related to phenolics, anthocyanins, flavonols, phenolic acids (non-flavonoid polyphenolic compounds), carotenoids, and other compounds as previously reported [17]. These data are supported by previous results showing that ROS production and cytotoxicity caused by airborne particles are attenuated by treatment with epigallocatechin gallate, a phenolic antioxidant found in a number of plants such as green and black teas [18]. These findings also, therefore, suggest that polyphenol-rich tart cherries are potential antioxidant prophylactic agents that can prevent oxidative stress-related diseases that affect human epidermal keratinocytes when they are exposed to fine dust.

Mitogen-activated protein kinases (MAPKs) involving ERKs, p38 MAPK, and JNKs are interesting candidates as downstream mediators of ROS. Given that ROS constitute the major regulators of apoptosis via regulation of MAPK signaling pathways [19], our data demonstrate that tart cherry abrogates the MAPK phosphorylation elicited by ROS to suppress the apoptotic signaling pathway mediated by the mitochondria in PM_10_-treated HaCaT cells. Our findings are supported further by the authors who revealed that MAPK is critical for the cell viability triggered by intracellular free radicals during exposure to air pollutants such as benzo[a]pyrene, cadmium, and acrolein [20,21,22]. Thus, our results suggest that the functional effect of tart cherry on the phosphorylation of ERK and p38 MAPK plays a key role in blocking the keratinocyte apoptotic signaling pathway elicited by PM_10_. Indeed, it was reported that PM exposure results in the phosphorylation of ERK and p38 MAPK in bronchial epithelial cells [23]. Hence, ERK and p38 MAPK inactivation by food phytochemicals may effectively prevent cell death as caused by various air pollutants, suggesting that ERK and p38 MAPK are major modulators and potential targets for the keratinocyte cytotoxicity caused by fine dust. These results collectively demonstrate that tart cherry has the ability to protect against apoptotic cell death by blocking the phosphorylation of the ERK and p38 MAPK pathways induced by PM_10_.

Next, we tried to understand the mechanism by which ROS and the ERK/p38MAPK pathway are associated with other molecular events closely linked to keratinocyte apoptosis induced by PM_10_. NF-κB, a redox-sensitive transcription factor, plays an important role in the gene transcription of the apoptotic signaling pathway in many epithelial cells when during exposure to various environmental contaminants, including fluoride, insecticides, and PM [24,25,26]. Indeed, it has been reported that NF-κB transcriptionally mediates the expression levels of many pro-inflammatory regulators and apoptotic genes in skin inflammation and damage [27]. The present results indicate that PM_10_ induces the phosphorylation of NF-κB as mediated by IκBα degradation during the promotion of keratinocyte apoptosis. In contrast, we found that tart cherry has an inhibitory effect on NF-κB mediated by ERK and p38 MAPK, which is necessary for the signaling events triggered by PM_10_ during the augmentation of apoptosis. Regarding the role of ERK and p38 MAPK on the phosphorylation of NF-κB, it was previously reported that the ERK and p38 MAPK pathways stimulated by ROS potentiate the transcriptional activities of NF-κB to enhance apoptotic genes’ expression [28,29]. Hence, these results imply that ROS initiated by PM_10_ have a significant role in enhancing the NF-κB pathway via the activation of ERK and p38 MAPK. Together, these observations demonstrate that ROS induced by PM_10_ can activate ERK, p38 MAPK, and NF-κB, whereas tart cherry blocks this activation of apoptosis-related signaling events in human epidermal keratinocytes.

Finally, we have shown that tart cherry significantly restores the levels of Bax and Bcl-2 affected by PM_10_. Having shown that fine dust induces oxidative stress and, thus, stimulates apoptotic cell death mediated by mitochondria via Bax oligomerization, Bax has been proven as a major determining factor for apoptotic susceptibility [30,31,32]. Moreover, earlier work indicated that increased NF-κB activity induces the transcription of many apoptotic genes, including Bax and Bcl-2, in response to butyric acid [33]. The mitochondrial translocation of Bax induces oligomer formation and mitochondrial membrane permeabilization, augmenting the mitochondrial cytochrome c release and caspase-9 activation that are required for caspase-3 activation [34,35]. On the other hand, Bcl-2 is localized to the mitochondrial outer membrane, where it plays a functional role in enhancing the survival and abrogating the activation of pro-apoptotic proteins. Consistently, we showed that tart cherry normalizes the levels of Bax and Bcl-2 triggered by PM_10_, representing unique downstream events of NF-κB activation in the mitochondrial apoptotic pathway related to mitochondrial ROS and caspase-3 activation.

## 5. Conclusions

The results of this study imply that tart cherry has the unique function of blocking the cellular mechanism caused by PM_10_, thus preventing the activation of the mitochondrial apoptotic pathway in human epidermal keratinocytes. These findings, therefore, highlight the relevance of a novel action of polyphenol-rich tart cherries in preventing the skin oxidative stress that leads to senescence and inflammatory diseases caused by FD. Moreover, our results provide an important insight into the potential for the development of therapeutic strategies and agents for improving the skin barrier functions injured by the natural and the anthropogenic dust sources.

## Figures and Tables

**Table 1:** The analysis condition of chlorogenic acid, quercetin, and kaempferol. FA—formic acid.

Time (min)	0.1% FA/Water (%)	0.1% FA/Acetonitrile (%)
0	98	2
1.0	98	2
2.0	90	10
4.0	70	30
7.0	50	50
9.0	30	70
11.0	10	90
13.0	0	100
14.0	98	2
16.0	98	2

**Table 2:** Contents of the compounds in tart cherry analyzed by UPLC.

Compound	Area (mV × sec)	Height (mm)	Content (mg/L)
Chlorogenic acid	27,304 ± 117.5	4446 ± 19.1	20.90 ± 0.18
Quercetin	37,875 ± 1708.5	5906 ± 266.4	8.20 ± 0.74
Kaempferol	21,373 ± 280	3406 ± 44.7	4.57 ± 0.12

## Data Availability

Data available on request due to restrictions eg privacy or ethical. The data presented in this study are available on request from the corresponding author.

## Abbreviations

Bax: Bcl-2 associated X protein; ERK: Extracellular signal-regulated kinase; MAPKs: Mitogen-activated protein kinases; NAC: *N*-acetylcysteine; NFC: Not from concentrate; NF-κB: Nuclear factor-kappa B; PM_10_: Fine particulate matter; ROS: Reactive oxygen species; UPLC: Ultra performance liquid chromatography.
